# Supplementary material for: Identification of a novel conserved signaling motif in CD200 receptor required for its inhibitory function
Source: PLoS One. 2021 Mar 29;16(3):e0244770. doi: 10.1371/journal.pone.0244770 (PMC8007030; doi:10.1371/journal.pone.0244770)
Supplement: S1 Table — A single sequence per species was used. (DOCX) [file pone.0244770.s004.docx]

| **Classes** | **Phylogenetic**  **tree** | **Overall conservation logo** | **Class specific conservation logos** |
| --- | --- | --- | --- |
| **Mammals** | 48 | 57 | 57 |
| **Birds** | 13 | 52 | 52 |
| **Bony fish** | 9 | 53 | 53 |
| **Reptiles** | 14 | 14 | n.a. |
| **Amphibians** | 3 | 3 | n.a. |
| **Total** | 87 | 179 | 162 |

**S1 Table.** **Number of species used per animal class for the phylogenetic tree (Fig 2A) and conservation logos (Fig 2B-E).**

A single sequence per species was used.
