# Supplementary material for: Identification of a novel conserved signaling motif in CD200 receptor required for its inhibitory function
Source: PLoS One. 2021 Mar 29;16(3):e0244770. doi: 10.1371/journal.pone.0244770 (PMC8007030; doi:10.1371/journal.pone.0244770)
Supplement: S2 Table — TR = Truncated. (DOCX) [file pone.0244770.s005.docx]

|  | **WT** | **TR** | **Y267F** | **Y286F** | **Y289F** | **Y297F** | **279**  **TTST** | **P285A** | **K292A** |
| --- | --- | --- | --- | --- | --- | --- | --- | --- | --- |
| **p-Erk** | 18 | 17 | 18 | 8 | 8 | 6 | 18 | 18 | 17 |
| **p-Akt308** | 14 | 14 | 14 | 10 | 10 | 8 | 14 | 14 | 12 |
| **p-Akt473** | 18 | 18 | 18 | 10 | 10 | 8 | 18 | 18 | 18 |
| **p-rpS6** | 14 | 14 | 13 | 6 | 6 | 6 | 13 | 14 | 14 |
| **IL-8** | 21 | 22 | 22 | 11 | 16 | 14 | 22 | 22 | 22 |

**S2 Table. Numbers of individual experiments per CD200R mutant and per residue shown in Fig 4.**

TR=Truncated.
