# Supplementary material for: Identification of a novel conserved signaling motif in CD200 receptor required for its inhibitory function
Source: PLoS One. 2021 Mar 29;16(3):e0244770. doi: 10.1371/journal.pone.0244770 (PMC8007030; doi:10.1371/journal.pone.0244770)
Supplement: Attachment — Submitted filename: Respons to Reviewers.docx [file pone.0244770.s007.docx]

Point-to-point response

Journal Requirements

When submitting your revision, we need you to address these additional requirements.

1. Please ensure that your manuscript meets PLOS ONE's style requirements, including those for file naming. The PLOS ONE style templates can be found at

https://journals.plos.org/plosone/s/file?id=wjVg/PLOSOne_formatting_sample_main_body.pdf and

https://journals.plos.org/plosone/s/file?id=ba62/PLOSOne_formatting_sample_title_authors_affiliations.pdf

**Response:** We changed the manuscript according to PLOS ONE's style requirements.

2. We note that you have stated that you will provide repository information for your data at acceptance. Should your manuscript be accepted for publication, we will hold it until you provide the relevant accession numbers or DOIs necessary to access your data. If you wish to make changes to your Data Availability statement, please describe these changes in your cover letter and we will update your Data Availability statement to reflect the information you provide.

**Response:** Our raw data and calculations are added to supplementary data file “S1 Data”.

3. Please include captions for your Supporting Information files at the end of your manuscript, and update any in-text citations to match accordingly. Please see our Supporting Information guidelines for more information: http://journals.plos.org/plosone/s/supporting-information.

**Response:** Captions for the Supporting Information files are now included at the end of our manuscript, and in-text citations are updated accordingly.

Review Comments to the Author

Reviewer #1: This work presents the significance of NPxY-motif in CD200R using the relatively new flow cytometry technology to make an effective record for visualization of research details. The authors demonstrate the step-by-step selecting the residue of interest in the NPxY motif, introducing the substitution, and effect of the single amino acid change in the human cell line.

1. To support Figure 1 result, I would recommend including prediction result of pathogenic effect with the substitution using public In-Silico analysis tools i.e., PANTHER, PROVEAN, PolyPhen2, SNAP2, or Mutation Assessor.

**Response:** We have included new *in silico* analysis as suggested. We took the intracellular tail of human CD200R used in the conservation plots and substituted every amino acid individually. With these substitutions we ran PROVEAN and PANTHER analysis. The results can be found in S3 File. We added the following text to the manuscript:

Page 4, line 52-53: “*In silico* predictions of possibly damaging amino acid substitution in the intracellular tail of human CD200R were generated using PROVEAN (5) and PANTHER (6).”

Page 12, line 218-220: “Additionally, PROVEAN (5) *in* *silico* analysis considered the substitutions N294A and P300A deleterious, while PANTHER (6) *in* *silico* analysis predicted the substitutions P285A, Y286F, Y289F and Y297F to be potentially damaging (S3 File).”

2. Line 210 mentioned that the different expression levels between each mutant. However, describing the method of how to normalize the difference is not sufficient.

**Response:** To make sure the results of our phosflow analysis was not affected by CD200R expression differences, during the analysis of these data, we set a CD200R expression gate to select for similar expression of CD200R in the cells analysed. See also the response to question 3 of Reviewer#2.

3.Line 318-323. These statements need to be rewritten with scientific interpretation of data reflecting the rate of the positive result.

**Response:** Unfortunately we do not understand what the reviewer asks from us. We assumed the text was unclear, and hope we have clarified what we meant to convey.

Page 16, line 347-356: “For phosflow analysis we corrected for differences in CD200R expression during the analysis of the flow cytometry data, which was not possible for IL-8 secretion. However, the effects of the mutations show a similar pattern in Erk phosphorylation and LPS-induced IL-8 secretion. CD200R-mediated inhibition of Erk was previously shown to be essential for inhibition of IL-8 (7). Therefore, we do not think (minor) expression differences have a major impact on our conclusion. For reasons we do not understand, both phosflow analysis and the IL-8 secretion measurements showed variation between experiments. However, as the number of experiments is high, we believe that the mean values are representative for the real biological situation.”

Reviewer #2: The authors identified novel conserved motifs on the cytoplasmic tail of CD200R. To my understanding, they ran the NCBI protein BLAST among different species. They made point mutations on these conserved motifs to test their roles on CD200R inhibitory function. This manuscript should be revised before accepted for publication.

1. This reviewer finds the abstract on the cover page differently compared to lines 14 – 28. I do not know if this is allowed in the editorial policy of PLOS ONE.

**Response:** Thank you for noticing. The correct abstract is the one in the manuscript, and we have updated the submitted files accordingly.

2. Introduction. I like to see more writings about the mechanism of CD200 and CD200R interaction in the immune response context. I will elaborate and place Figure 3A in the introduction section. For the general audience, the authors must provide CD200R involvement in 'inhibitory signaling.' I understood this manuscript is about the inhibition of inhibitory signaling. Readers need to know how the wild type cytoplasmic tail inhibits the downstream signaling before approaching the primary data.

**Response:** We thank the reviewer for this suggestion and have updated the introduction accordingly. We have moved the figure with a model for CD200R signaling to the introduction, and mentioned the contributions of CD200R to (suppressing) immunity in the introduction.

Page 8, line 134-142 mentions the contributions of CD200R to (suppressing) immunity.

Page 9, line 153-162 describes the model for CD200R signaling in the introduction. This subsequently led to some changes in the text of the result section, see page 13, line 256-259.

Page 9, line 179-183 includes the figure legend.

3. Figure 2B needs more explanation. Figure 2B needs labels on the X- and Y-axis, the relationship among these three plots, and what those peaks between control and mutants mean.

**Response:** We clarified the labelling on the X- and Y-axis and explained the relationship among the three plots more clearly. From left to right we first selected live cells based on forward and side scatter, then from these live cells we selected for single cells and subsequently we show CD200R expression. We have added labels to the arrows to clarify this.

In this figure we included the gating strategy for phosflow analysis. There, we subsequently set a gate based on CD200R expression to ensure we only analyzed cells with similar amounts of CD200R expression. See also the response to question 2 of Reviewer#1.

In the figure legend the following text is added (page 13, line 249-252): “First the population of live cells was selected. From these live cells, single cells were selected and from these single cells CD200R expressing cells were selected for phosflow analysis. SSC-A: side scatter area; FSC-A: forward scatter area; FSC-W: forward scatter width.”

4. Line 210. We need to explain how to make sure all mutant proteins are expressed at the cell surface from Figure 2B.

**Response:** See the response to question 3 above and to question 2 of Reviewer#1.

5. Line 230-232 need more explanation about how to correct for differences in CD200R expression between mutants. How does the CD200R expression gate was set for Phosflow analysis?

**Response:** See the response to question 3 above and to question 2 of Reviewer#1. We extended the text as follows (page 13, line 260-262): “We only analyzed cells with similar CD200R expression by applying an CD200R-expression gate in our phosflow analysis. This gate was set as indicated in figure 3B.”

6. Line 274: what does ‘low importance’ mean?

**Response:** We clarified this (page 15, lines 305-306): “In line with literature, we found Y286F and Y289F to be about 50% less efficient in CD200R mediated inhibition of LPS-induced IL-8 secretion (7, 41).”
